# Needle in a Haystack—Parathyroid Gland in a 10-Day Old Infant: A Case Report and Literature Review

**DOI:** 10.5402/2011/678070

**Published:** 2011-08-03

**Authors:** Adel Ismail, Tariq O. Abbas, Fawziya Al-Khalaf

**Affiliations:** ^1^Pediatric Surgery Department, Hamad General Hospital, P.O. Box 3050, Doha, Qatar; ^2^Pediatrics Department, Hamad General Hospital, P.O. Box 3050, Doha, Qatar

## Abstract

Neonatal severe primary hyperparathyroidism (NSPHT) is a rare autosomal recessive disorder of calcium homeostasis. It presents shortly after birth and is characterized by striking hyperparathyroidism, marked hypercalcemia, and hyperparathyroid bone disease. It is caused by mutations of the calcium-sensing receptor (CASR), the ionized calcium sensor for the parathyroid cells, the parafollicular thyroid C cells, and the kidney epithelium, as well as cells in bone and intestine. Without early intervention, which frequently requires surgical removal of the hyperplastic parathyroids, the patients often succumb to complications of hypercalcemia and respiratory failure. Finding the parathyroid gland in small neonates is not an easy task. Here we report on a patient with neonatal hyperparathyroidism who was treated by total parathyroidectomy and discuss the various ways of helping to find the parathyroid glands during surgery at this young age.

## 1. Case Report

The patient is a 3.2 Kg female the product of uneventful 38-week gestation. The parents are first cousins from Sri Lanka and were unaware of any similar cases in their extended family. APGAR scores were 8 and 9 at 1 and 5 min, respectively. The newborn was well for the first few days of life, but on the 7th day of life, she became progressively more hypoactive and fed poorly. She was transferred to a pediatric intensive care unit with a presumptive diagnosis of neonatal sepsis.

On physical examination the weight was 2.2 Kg, with a loss of 1 Kg since birth. The infant was hypoactive and dehydrated. Blood pressure was 85/55 mmHg, with generalized hypotonia. Biochemical analysis showed hypercalcemia (corrected calcium, 4 mmol/L (normal <2.73 mmol/L); total calcium, 6 mmol/L). Ionized serum calcium ranged between 1.85 and 2.05 mmol/L (normal <1.23 mmol/L), and serum phosphate was as low at 0.42 mmol/L (normal >1.45 mmol/L). Serum alkaline phosphatase activity was high (518 IU/L; normal <420 IU/L). Both serum creatinine and urea were high at 133 umol/L (35 : 53 umol/L0) and 38 mmol/L (1.4 : 5 mmol/L), respectively. Serum electrolytes (sodium 160 mmol/L; potassium 3.0 mmol/L; chloride 126 mmol/L; and bicarbonate 21 mmol/L), all compatible with dehydration and prerenal failure.

The clinical diagnosis of NSHPT was based on the early onset, life-threatening hypercalcemia, and the radiologic evidence of hyperparathyroidism. On the second day of admission, the parathyroid hormone level was 573 ng/L (normal, 15–65 ng/L). The urine calcium/creatinine index (both measured in millimoles) was 8.3 (normal <0.4). The serum 25-hydroxycholecalciferol was 23 nmol/L (normal values 75–200 nmol/L). Skeletal survey revealed generalized skeletal undermineralization, with evidence of subperiosteal bone resorption. Metaphyses of the long bones showed marked destruction with cupping and fraying and a metaphyseal fracture of the left femur was observed. The mandible and skull were normal. 

The patient's hypercalcemia was managed initially with hyperhydration, furosemide, and calcitonin with minimal effect. She also received maintenance dose of 400 IU/day of vitamin D3. On day 2, intravenous pamidronate treatment (20 mg/m^2^, equivalent 3 mg) was started, without evidence of adverse reaction. In particular, there was no hypomagnesemia, vomiting, or respiratory distress, and no evidence for lymphocytopenia or a febrile reaction. The corrected serum calcium dropped to a minimum of 3.18 mmol/L, but the PTH level increased to 1,900 ng/L. 

An ultrasound of the neck failed to demonstrate enlarged parathyroid glands, and renal ultrasound showed no evidence of renal stones or nephrocalcinosis. Sestamibi scintigraphy showed no evidence of ectopic parathyroid tissue.

At age 10 days, the infant underwent exploratory surgery of the neck. Intraoperatively, 0.1 mg/kg of methylene blue was given very slowly intravenously to help in localizing the parathyroid glands. The parathyroid glands although very tiny at this age, but taken the methylene blue rendering them relatively easy to indentify. Two parathyroid glands were located on the left side, but on the right side, only the upper parathyroid could be identified. A piece of tissue considered as possibly right lower parathyroid was negative on subsequent histopathologic analysis. The three excised parathyroid were confirmed histologically to be hyperplastic parathyroid glands. On effect, we were left with remaining right lower parathyroid gland unaccounted for.

During surgery and immediately after, the serum PTH dropped rapidly from a preoperative high of 1,383 ng/L to 791, 395, 324, and 310 ng/L at hourly intervals after removal of the glands. Postoperatively, serum PTH levels were 426, 432, and 452 ng/L, respectively, at weekly intervals. By day 3 post-op, the patient developed transient asymptomatic hypocalcemia (corrected calcium was 1.8 mmol/L and phosphate was 1.0 mmol/L) despite a PTH level of 426 ng/L. Treatment with oral alphacalcidiol (0.1 ug/kg/day) and calcium (40 mg/kg/day elemental Ca) was started. Three weeks and 3 months postoperatively, the calcium/urine ratio dropped to 0.2 then it became 0.16, respectively.

Two and half months later, hyperparathyroidism (PTH = 305 ng/L) and hypercalcemia (total calcium = 4.0 mmol/L) had recurred, although the 25(OH) D had increased to 80 nmol/L (normal, 75–200 nmol/L). 

At the age of 3 months, it was decided to reexplore the neck to identify the fourth parathyroid gland. No imaging was done in view of the failure of this imaging to be helpful before the first operation. At re-operation, we looked for this gland at all the possible sites but could not find this missing gland. As we are aware that a parathyroid could be intrathymic or even intrathyroid so it was decided to do empiric thymectomy and right hemithroidectomy, as it is the side where this missing parathyroid is sited. Fortunately, histopathologic examination of the right hemithyroid revealed an intrathyroid parathyroid gland.

The serum PTH dropped from 407 ng/L preoperatively to 6 ng/L and 3 ng/L on days 1 and 3 after surgery, respectively. The calcium level (4 mmol/L preoperatively) normalized to 2.3 mmol/L within a day after surgery.

Oral calcium (30 mg/kg/d of elemental Ca every 6 h) and 1 alpha calcidiol (0.25 *μ*g daily) were started. The serum levels of free thyroxine (fT4) were 18, 14, 16, 17 pmol/L (10.23–23.17 pmol/L) and thyroid-stimulating hormone levels were 1.18, 6, 8, and 4.6 mU/L (0.3–5 mIU/L) at day 1, month 1, month 3, and month 5, respectively, after surgery. A repeat skeletal survey 3 months after final surgery showed increased bone density, healing of the metaphyseal fracture, and resolution of the subperiosteal resorption. Two months after surgery, the patient had asymptomatic hypocalcemia (total calcium: 1.2 mmol/L) during a clinic visit. She was maintained on oral elemental calcium (100 mg/kg/d) and alphacalcidiol (0.2 *μ*g/kg/d) in order to correct hypocalcemia. Her serum magnesium was 0.65 mmol/L (normal, 0.65–1.0 mmol/L). No thyroid dysfunction was detected postsurgically. She did not have any hypocalcemia afterwards and phosphorus level after 2 years was 2.58 mg/dL.

## 2. Discussion

NSHPT can present at any time within the first 6 months of life, but is often discovered in the first few weeks postnatally. Affected infants have severe, symptomatic, PTH-dependent hypercalcemia, along with the bony changes of severe hyperparathyroidism. They often exhibit polyuria, dehydration, and hypotonia associated with a history of failure to thrive. Early diagnosis is critical as untreated NSHPT can have a devastating neurodevelopmental effects.

Although some patients have been treated with acute medical management and subtotal parathyroidectomy, total parathyroidectomy with or without autotransplantation is the recommended line of management at the moment.

The surgical management of neonatal hyperparathyroidism was first reported in 1964, and the urgency of doing this was recognized shortly after. Initially, subtotal parathyroidectomy was the procedure of choice, but owing to a significant rate of recurrence, total parathyroidectomy was adopted. Total parathyroidectomy proved to be effective in controlling the disease with minimal morbidity, but lifelong calcium and vitamin D supplements were required. Following the experience with adult hyperparathyroidism, reports of total parathyroidectomy with heterotopic autotransplantation appeared in the literature. This is to avoid the lifelong replacement therapy. Following autotransplantation, a failure rate of 6% has been reported, and 33% are expected to have graft-dependent hypercalcemia.

There are different modalities that have been used to localize the hyperfunctioning parathyroid glands (Table 1).

While there are known diagnostic means in adults that help localize the parathyroid adenomas or hyperplastic glands before surgery, for example, the combination of 99mTc-sestamibi/99mTc-pertechnetate subtraction scintigraphy (SS) and high-resolution neck ultrasonography (US), this was found not to be true in children. 

Al-Shanafey et al. [2] showed that preoperative localization studies are not helpful. This was recommended by others too.

In the adult patients it is not always easy to find the four parathyroid glands during surgery. This is because of the variable locations and the small size of these glands. The parathyroid glands are the most inconsistent, in location, among all the endocrine glands in humans. So, knowledge of the possible locations of these glands is essential before embarking on performing parathyroidectomy.

The parathyroid glands, typically four in number, develop from the dorsal extremities of the third and fourth pharyngeal (branchial) pouches and are sometimes therefore designated as parathyroid glands III and parathyroid glands IV. In the course of their development, the parathyroids derived from the third pouches soon become associated with the thymus, which develops primarily from these pouches, while those from the fourth pouches become associated with the developing thyroid gland. 

As the thyroid and thymus, with their associated parathyroid glands, move caudally from the region in which they originate, the thymus normally descends beyond the level at which the thyroid halts. The parathyroids derived from the third branchial pouches are therefore typically carried father than those derived from the fourth branchial pouches. Thus the parathyroids from the fourth pouches are typically located more cranially on the thyroid gland and are called superior parathyroid glands, while those derived from the third pouches are typically freed from the thymus and become associated with the thyroid gland toward its lower pole, becoming the inferior parathyroid glands. 

**Figure 1:**
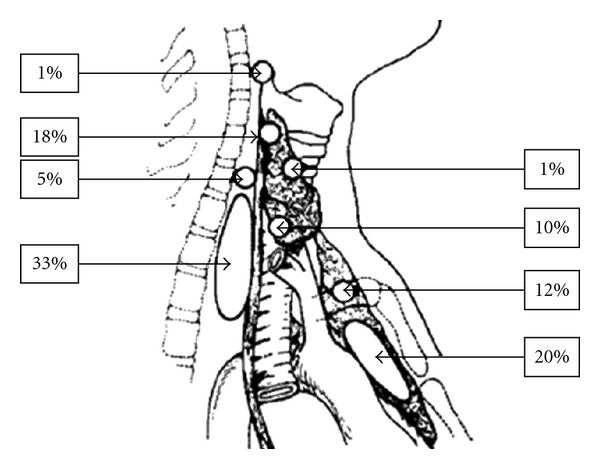
Anatomic locations of abnormal parathyroid glands found at reoperation by single group [1].

The superior parathyroid tends to be less variable than the inferior glands and they are generally found in the posterior surface of the thyroid gland. The lower parathyroids have a longer and more complex journey to reach their final destination caudal to the superior ones and can have more variable sites. Parathyroids can be found also anywhere along the tracheoesophageal groove (Figure 1).

Beside these various sites, parathyroid are also known to exist in some rare sites, for example, the thymus or intrathyroid. Knowledge of this fact guided us during surgery on the course to take. To identify the glands during surgery, Kuriloff and sanborn 2004 [3] found that intraoperative methylene blue infusion to be safe and effective in rapid identification of parathyroid tissue.

On conclusion neonatal hyperparathyroidism is a rare congenital anomaly which is life threatening. Bilateral surgical exploration is the definitive mode for their detection and removal. Intraoperative methylene blue infusion is helpful in localizing the parathyroid glands. These glands could exist in some rare sites, for example, the thymus or intrathyroid.

## Figures and Tables

**Table 1:** Parathyroid gland localization modalities.

Non invasive preoperative	
(i) Ultrasound	
(ii) MRI	
(iii) CT	
(iv) Thallium-technetium scintigraphy	
(v) Technetium-99 Sestamibi scintigraphy	
(vi) Radioiodine or technetium thyroid scan	

Invasive preoperative	

(i) Selective arteriography or digital subtraction	
(ii) angiography	
(iii) Selective venous sampling	
(iv) Fine-needle aspiration	

Intraoperative	

(i) Ultrasonography	
(ii) Methylene blue injection	
(iii) Quick parathyroid hormone measurements	
